# How Do You Identify m^6^ A Methylation in Transcriptomes at High Resolution? A Comparison of Recent Datasets

**DOI:** 10.3389/fgene.2020.00398

**Published:** 2020-05-20

**Authors:** Charlotte Capitanchik, Patrick Toolan-Kerr, Nicholas M. Luscombe, Jernej Ule

**Affiliations:** ^1^The Francis Crick Institute, London, United Kingdom; ^2^Department of Neuromuscular Diseases, UCL Queen Square Institute of Neurology, London, United Kingdom; ^3^Department of Genetics, Environment and Evolution, UCL Genetics Institute, London, United Kingdom; ^4^Okinawa Institute of Science and Technology Graduate University, Okinawa, Japan

**Keywords:** RNA, N6-methyladenosine, m^6^A, epitranscriptomics, bioinformatics

## Abstract

A flurry of methods has been developed in recent years to identify N6-methyladenosine (m^6^A) sites across transcriptomes at high resolution. This raises the need to understand both the common features and those that are unique to each method. Here, we complement the analyses presented in the original papers by reviewing their various technical aspects and comparing the overlap between m^6^A-methylated messenger RNAs (mRNAs) identified by each. Specifically, we examine eight different methods that identify m^6^A sites in human cells with high resolution: two antibody-based crosslinking and immunoprecipitation (CLIP) approaches, two using endoribonuclease MazF, one based on deamination, two using Nanopore direct RNA sequencing, and finally, one based on computational predictions. We contrast the respective datasets and discuss the challenges in interpreting the overlap between them, including a prominent expression bias in detected genes. This overview will help guide researchers in making informed choices about using the available data and assist with the design of future experiments to expand our understanding of m^6^A and its regulation.

## Introduction

N6-methyladenosine (m^6^A) is the most abundant internal modification of messenger RNA (mRNA), occurring ubiquitously across the tree of life. In mammals, m^6^A is thought to be deposited cotranscriptionally by the METTL3–METTL14–WTAP complex, with METTL3 being the catalytically active methyltransferase (Ke et al., 2017; Bertero et al., 2018). There is a strong enrichment for this modification within a degenerate DRACH sequence context (D = A, G, or U; R = A or G; H = A, C, or U), with early chromatographic studies suggesting a core RAC motif (Wei and Moss, 1977). The knockout of *METTL3* is embryonic lethal in mice, indicating its critical role in regulating mammalian development (Geula et al., 2015): the modification is implicated in diverse cellular processes such as differentiation, meiosis, circadian rhythms, and proliferation in cancer (Fustin et al., 2013; Schwartz et al., 2013; Batista et al., 2014; Geula et al., 2015; Cui et al., 2017). As a posttranscriptional regulator, m^6^A is especially interesting in the context of neurons, where it can potentially regulate localized translation (Merkurjev et al., 2018; Shi et al., 2018). The best understood mechanism of m^6^A function is via the direct binding of YTH domain proteins, which target m^6^A-containing transcripts for nuclear export, translation, and decay (reviewed in Patil et al., 2018).

To develop a detailed understanding of how m^6^A dictates mRNA fate, we need to determine exactly which mRNA sites are m^6^A modified in a given biological system. To this end, high-throughput approaches have been developed to map m^6^A transcriptome-wide (Table 1). However, the modification presents significant challenges, as reverse transcription of native m^6^A nucleotides using common reverse transcriptases does not yield a specific mutational or truncation-based signature, unlike other RNA modifications.

**TABLE 1:** Single nucleotide resolution, transcriptome-wide methods for detecting m^6^A.

**Method type**	**Method**	**Cell lines (human)**	**Strengths**	**Weaknesses**	**Motif restriction?**	**Diagnostic signature**	**UMI**	**RNA selection**	**References and (data access)**
Antibody based	miCLIP	HEK293 MOLM13	• High throughput, can be used to assess multiple conditions • RNA can be taken from any source as crosslinking occurs *in vitro* • Reproducible data	• Difficult to correct for nonspecific antibody binding • Requires UV crosslinker • Complex library preparation • Requires high amounts of input material	DRACH	Truncations and C → T mutations	Yes	Total RNA and poly(A) selected available	Linder et al., 2015; Vu et al., 2017 (GSE98623)
	m^6^A-CLIP	A549 CD8+ T cells HeLa			RRACU/RAC	Truncations and mutations (substitutions and deletions)	Yes	poly(A) HeLa—ribo0, poly(A), nucleoplasm, chromatin	Ke et al., 2015 (GSE71154); Ke et al., 2017 (GSE86336)
MazF enzyme based	MAZTER-seq	HEK293T	• Generates stoichiometric data • Semiquantitative output	• Can only detect sites in ACA sequence context • Sequence-specific biases in enzyme cutting efficiency • Complex bioinformatics analysis	ACA	Enzymatic cleavage efficiency, measured as truncations vs. read-through	No	poly(A)	Garcia-Campos et al., 2019
	m^6^A-REF-seq	HEK293T			ACA		No	poly(A)	Zhang Z. et al., 2019
Fusion domain based	DART-seq	HEK293T	• Low RNA input • Simple library preparation	• Biases in background APOBEC1 targeting • Mapping is limited to YTH-recognized sites • Resolution is low compared to CLIP methods • Must express fusion construct *in vivo* for maximum efficiency	Mutation site must be C → U	C → U mutations	No	None	Meyer, 2019
*In silico* prediction	WHISTLE	Any	• Can predict m^6^A sites in any gene, regardless of expression	• Trains based on CLIP datasets, so will learn CLIP biases	RRACH	Truncations and mutations	Yes	poly(A)	Chen et al., 2019 (http://180.208.58.19/whistle/download.html)
Direct RNA sequencing by Nanopore	MINES	HEK293	• Potential for measuring stoichiometry of sites and combinatorial modification dynamics (although currently not systematically implemented)	• Trains based on CLIP datasets, so will learn CLIP biases	RGACH	Tombo’s fraction modified values and coverage files	NA	poly(A)	Lorenz et al., 2019
	NanoCompore	MOLM13	• Can detect other modifications as well as m^6^A • Potential for measuring stoichiometry of sites and combinatorial modification dynamics (although currently not systematically implemented)	• Currently low throughput • High input requirements • Requires a low or no methylation control, which might be difficult to obtain	No	Difference in k-mer current intensity and dwell time in pore between WT and METTL3 KD control	NA	poly(A)	Leger et al., 2019

Here, we provide a brief technical overview of the major methods to identify m^6^A transcriptome-wide at single nucleotide, or near single nucleotide, resolution highlighting the respective advantages and drawbacks of each method. Furthermore, by comparing genes identified by each method, we begin to explore their resulting datasets.

### Antibody-Based Methods

**FIGURE 1:**
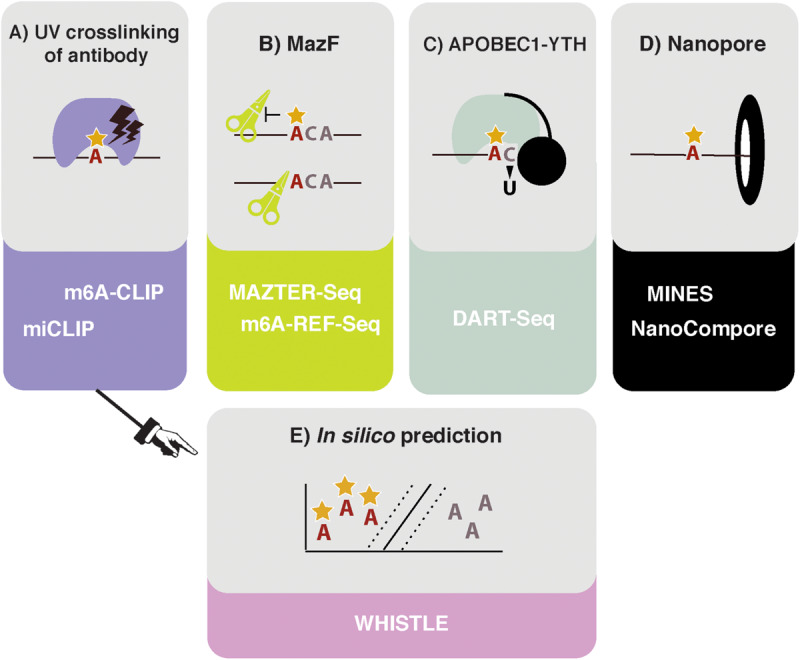
High throughput methods to detect or predict m^6^A in transcriptomes. **(A)** Crosslinking and immunoprecipitation (CLIP) methods involve UV crosslinking of the m^6^A antibody to purified RNA. m^6^A-CLIP and miCLIP differ in the antibodies used, complementary DNA (cDNA) library preparation, and computational processing, among other differences. **(B)** MazF *Escherichia coli* endoribonuclease preferentially cuts at nonmethylated ACA sites. This forms the basis of MAZTER-seq and m^6^A-REF-seq. **(C)** DART-seq expresses an APOBEC1-YTH fusion protein. The YTH domain targets APOBEC1 to m^6^A sites, where it deaminates surrounding cytosines to uracil. **(D)** Direct RNA sequencing with Nanopore technologies facilitates detection of m^6^A due to differences in ionic current intensities between A- and m^6^A-containing sequences and dwell time in the pore. Methods differ by how these signals are deconvolved. m^6^A identification using nanopore sequencing (MINES) is a combination of four random forest models, pretrained using CLIP m^6^A sites as true positives. NanoCompore relies on a comparison in signal between two conditions, for example wild type (WT) and METTL3 knockdown, or *in vivo* RNA vs. nonmodified *in vitro* transcribed RNA. **(E)** *In silico* prediction of m^6^A sites is performed by WHISTLE, a support vector machine algorithm that uses miCLIP and m^6^A-CLIP sites as training data.

The first described methods for transcriptome-wide profiling of m^6^A were m^6^A-seq and MeRIP-seq. These methods use an antibody for m^6^A to perform RNA immunoprecipitation, followed by next generation sequencing (NGS) (Dominissini et al., 2012; Meyer et al., 2012). However, the resolution of m^6^A-seq is limited to the size of RNA fragments, with no objective way of determining where in the fragment the modification occurred. Greater resolution was achieved by UV crosslinking the antibody to RNA, following the principles of the crosslinking and immunoprecipitation (CLIP) protocol (König et al., 2010). Such approaches were simultaneously developed in the laboratories of Samie Jaffrey and Robert Darnell, named miCLIP and m^6^A-CLIP, respectively (Figure 1A; Ke et al., 2015; Linder et al., 2015). Here, purified RNA is incubated *in vitro* with an m^6^A antibody. Following immunoprecipitation, the antibody is digested with proteinase K, leaving an amino acid adduct attached to the RNA base. During preparation of the complementary DNA (cDNA) library, the reverse transcriptase either reads through this crosslinked adduct, causing a substitution or deletion mutation, or is stopped, resulting in cDNA truncation. These signals can be analyzed computationally to identify the modification site at single nucleotide resolution (Haberman et al., 2017). The Jaffrey group found that antibodies differed in their propensities to introduce a mutation or truncation and in the positions of these signals in relation to the modified adenosine. The authors concluded that the polyclonal Abcam and Synaptic Systems antibodies were most efficient at immunoprecipitating and gave the most predictable mapping signatures; as a result, they remain the most commonly used antibodies in subsequent miCLIP publications.

N6-methyladenosine-crosslinking and immunoprecipitation is conceptually similar to miCLIP but requires preparation of multiple libraries and has so far exclusively used the Synaptic Systems antibody. Two sequencing libraries are prepared from the same sample: one using the MeRIP-seq approach to identify m^6^A-modified oligonucleotides and one using the miCLIP approach, which is then analyzed to identify both reverse transcription read-through and truncation events. These signals are then filtered to retain only those that overlap with peaks from the MeRIP-seq library. In this way, the authors claimed greater specificity in identifying true modification sites. The protocol differs from the miCLIP protocol in several additional ways; for example, size selection of RNA fragments prior to immunoprecipitation and a bromodeoxyuridine (BrdU) cDNA-purification approach. There are also differences in the starting RNA/antibody ratios—miCLIP uses an excess of RNA, whereas m^6^A-CLIP uses an excess of antibody.

A major drawback with these approaches is the promiscuity of m^6^A antibodies; for example, some interact with m^6^Am, which is found as the first nucleotide after the cap in certain mRNAs (Schwartz et al., 2013; Linder et al., 2015). Devising appropriate methods to eliminate false positives is challenging. Studies generally tackle this issue by only reporting sites found within the consensus DRACH motif or by perturbing methyltransferase activity. Neither is optimal: DRACH-only reporting prevents discovery of m^6^A in RAC or noncanonical motifs, whereas knockout or knockdown controls exclude sites that can be modified by another methyltransferase. Furthermore, disrupting the m^6^A machinery may introduce global changes in RNA abundance that are difficult to account for, except with the careful use of input libraries and spike-ins (Liu et al., 2020).

Finally, methods that depend on crosslink-induced mutations as the readout—as opposed to truncations—may be more susceptible to gene expression changes because higher read coverage is required to call sites. Additionally, for all strategies, the necessary integration of multiple control datasets (methyltransferase depletion, RNA input, etc.) increases the variance in the experimental design, reducing the statistical power to call sites. In summary, although antibody-based methods have been fundamental to paving the way for transcriptomic analysis of m^6^A and remain the most common way to survey the modification, issues with antibody specificity make orthogonal approaches desirable.

### Enzyme-Based Methods

In 2017, the MazF endoribonuclease was described, which cuts RNA within an ACA sequence motif, but with greater preference for ACA over m^6^A-CA sites (Imanishi et al., 2017). Thus, m^6^A-modified sites, usually present within a DRACH motif, can be detected as a reduction in MazF cleavage efficiency. Two new methods, MAZTER-seq and m^6^A-REF-seq (Figure 1B) developed by the laboratories of Schraga Schwartz and Guan-Zheng Luo, respectively, showed how this enzyme can be used to map m^6^A at single-nucleotide resolution (Garcia-Campos et al., 2019; Zhang Z. et al., 2019).

In both approaches, purified mRNA is treated with the MazF enzyme, leaving RNA fragments containing an ACA site at the 5′ end and finishing just before the next ACA motif within the transcript. After sequencing, any ACA sequences present within a read indicate an uncut and, therefore, modified site. The main advantage of this approach is that it can provide stoichiometric information on the m^6^A modification, based on the cut/uncut ratio of reads for every ACA site, something the antibody-based methods currently lack.

Nevertheless, due to the specific attributes of the MazF enzyme, careful quality control in calculating m^6^A stoichiometry is required. In MAZTER-seq, potential m^6^A sites are prefiltered to remove any ACA sequences that are too close to each other to be accurately measured. Furthermore, reads that do not begin and end within a cleaved ACA sequence are removed, as they could occur through random RNA fragmentation or nonspecific cutting. Finally, for a subset of analyses, ACA sites containing a G at the +3 position are removed, as this impairs MazF cleavage efficiency. The authors calculate that, theoretically, 25% of DRACH sites in yeast and 16% in mammals can be quantified using MAZTER-seq. In contrast, m^6^A-REF-seq does not apply filters based on incorrect read endings or calculations of the minimal ACA proximity; instead, ACA sites predicted to be in double-stranded RNA regions are discarded, as they are considered to alter cutting efficiency. Furthermore, for a site to be called, the authors require a decrease in the modification ratio >10% when the RNA is treated with the demethylase enzyme FTO.

In addition to calculating stoichiometric ratios of CLIP-annotated m^6^A sites, MAZTER-seq was used to identify previously unknown m^6^A sites. This was achieved by comparing cleavage efficiencies within DRACH motifs in three different control scenarios. The first was between WT and m^6^A methyltransferase deletion input libraries, the second was m^6^A-IP with the same strains, and the third, a comparison between input and m^6^A IP WT conditions. In this way, the authors classified all published sites into confidence groups and found a number of previously unannotated sites within the high-confidence groups. Crucially, this suggests that probable m^6^A sites have been missed by antibody-based methods.

MazF clearly enables valuable approaches to calculate m^6^A stoichiometry at a focused set of sites, validate previously identified m^6^A sites, and identify a number of novel sites. The limitation of the MazF enzyme to ACA sites and the extensive filtering requirements do mean, however, that these methods alone cannot provide a full transcriptome-wide map of m^6^A. Nonetheless, the careful work to identify and quantify the biases inherent in this system is of great value in developing high-confidence m^6^A maps and offers an important orthogonal method to other transcriptome-wide mapping approaches.

### Fusion Domain-Based Methods

DART-seq employs the *in vivo* expression of a YTH protein domain fused to the APOBEC1 enzyme (Figure 1C; Meyer, 2019). The YTH domain was identified in numerous studies as the major “reader” of the m^6^A modification (Zaccara et al., 2019), whereas the APOBEC1 enzyme deaminates cytosine to uracil, which can be detected as a mutation compared with a reference sequence. Thus, this construct allows deamination of cytosine residues in the vicinity of m^6^A sites recognized by YTH. Previous studies suggest that m^6^A is invariably followed by cytosine (Wei et al., 1976), raising the possibility of single-nucleotide resolution mapping, although in practice, more distant cytosines are also modified.

The most notable benefit is the low input requirements: libraries can be made with as little as 10 ng of total RNA as starting material. Additionally, as the YTH-APOBEC1 construct can be transiently expressed in cells, library preparation is much more straightforward than either the antibody- or enzyme-based methods, since no treatment of the RNA is required to identify the m^6^A signal following extraction. Owing to targeting by the major m^6^A reader, it is also possible that DART-seq will identify more functionally relevant m^6^A sites than other methods. One possible drawback is that the APOBEC1 enzyme displays sequence preferences: expressed alone, it modifies cytosine residues in the 3′ untranslated region (UTR), making it difficult to detect confidently in this region, while ∼70% of APOBEC1-only deaminated sites are preceded by an adenosine (Supplementary Figure 6C from Meyer, 2019), meaning that using APOBEC1 and APOBEC1-YTH mutant as a control is likely to result in false negatives.

### Direct Sequencing-Based Methods

Ideally, it would be possible to detect m^6^A via direct RNA sequencing. Pore-based sequencers measure changes in an ionic current as nucleic acids pass through a nanopore: information about changes in current and dwell time in the pore is used to identify the nucleotide in question. Several publications demonstrated that RNA modifications produce specific current and dwell time signals, suggesting nanopore-based methods could identify modified nucleotides in a high throughput manner (Figure 1D; Garalde et al., 2018; Workman et al., 2018; Smith et al., 2019). The potential benefits of this approach for mapping RNA modifications are huge, as stoichiometric and positional information of multiple modifications could be interpreted simultaneously. The reality of deconvolving the raw signal to infer m^6^A sites, however, is not straightforward.

The first application of the Oxford Nanopore technology (Nanopore) to detect m^6^A in a whole transcriptome examined yeast mRNA (Liu et al., 2019). The authors trained a support vector machine (SVM), called EpiNano, on Nanopore sequencing data of synthetic transcripts containing m^6^A residues in every possible 5-mer combination to identify the most informative signals that distinguish m^6^A from other nucleotides. Surprisingly, the raw current intensities alone were found to be poor predictors of methylation status; instead, the selected training features included mean per-base quality, mismatch frequency, and deletion frequency. The model achieved ∼90% prediction accuracy for the training dataset. It was then used to recover 363 previously identified, high-confidence m^6^A sites, previously identified using m^6^A-seq, which it was able to do with 87% accuracy.

An alternative approach, m^6^A identification using nanopore sequencing (MINES), was used to create the first Nanopore-based m^6^A transcriptome for humans (Lorenz et al., 2019). This method applied Tombo, a program that was previously developed to detect *de novo* modifications in Nanopore DNA-sequencing data based on base-calling errors (Oxford Nanopore Technologies, 2018). The authors trained random forest models using the Tombo modification values to classify the m^6^A status of four RGACH motifs. Those RGACH sites overlapping with HEK293 miCLIP and HeLa m^6^A-CLIP sites (Linder et al., 2015; Ke et al., 2017) were labeled as true positives in the training data, and the models achieved an average accuracy of 79%, representing 35% of m^6^A sites identified with CLIP-based methods (in part due to the motif restriction). The authors then predicted 13,034 novel RGACH m^6^A sites, which were validated by METTL3 knockdown.

A further approach is NanoCompore (Leger et al., 2019), which compares Nanopore signals between two datasets and therefore does not require a training dataset. Specifically, this is achieved by contrasting the median current intensities and dwell times of k-mers between the experiment and a control with perturbed modifications (e.g., wild type vs. knockdown, or *in vitro* modified vs. unmodified controls). To identify METTL3-dependent m^6^A sites, the authors processed polyA+-selected RNA sequencing data from wild-type and METTL3 short-hairpin RNA (shRNA) knockdown MOLM13 cells. NanoCompore is not restricted to m^6^A and can be readily extended to other modifications that have a reliable control. A major advantage is that it avoids being biased by the accuracy of previous mapping methods to train the models, as site identification is instead determined by the sensitivity to a specific modification enzyme. Of course, the dependence on a comparison between samples is a limitation, as reliable controls are currently unavailable for many modifications and biological systems, and specific sites or RNA species are often modified by distinct enzymes. As a result, there is probably a reduced risk of false-positive site assignment at the cost of sensitivity.

Finally, a simplified approach was recently published for the *Arabidopsis thaliana* transcriptome (Parker et al., 2020), in which the base-calling error rate was used as the sole parameter for identifying m^6^A sites. The authors compared the transcriptomes for a *vir-1* mutant, an Arabidopsis m^6^A methyltransferase, with a *vir-1* restored line, identifying ∼17,000 sites with an error rate twofold greater in the control line compared to mutant. Taking this approach 66% of identified m^6^A sites fell within five nucleotides of a miCLIP peak.

The above methods demonstrate that direct RNA sequencing can be used to detect m^6^A. A common limitation pertains to the resolution and accuracy of modification assignment for transcripts with low sequencing depth. However, with third-generation sequencing technologies developing rapidly, the benefits of using direct sequencing to map RNA modifications—such as the possibility of correlating modifications with other transcriptomic features within a single RNA molecule, and accurately calculating m^6^A stoichiometry genome-wide—are likely to push the boundaries of the field.

### *In silico* Prediction

Even in the best circumstances, experiments are still costly and time consuming to run and can only identify m^6^A sites that are present in the prepared sample. *In silico* prediction offers the potential of identifying all possible m^6^A sites (Figure 1E). However, algorithms rely on two critical factors: (i) the reliability of the training data and (ii) the ability to identify and encode relevant features indicating m^6^A presence into the model. Existing approaches either use SVMs (methyRNA—Chen et al., 2017; RNAMethPre—Xiang et al., 2016; WHISTLE—Chen et al., 2019) or random forest models (RF; SRAMP—Zhou et al., 2016) to classify whether or not an adenosine is modified. The benefits of a machine-learning model, over other modeling approaches, is that predictive features do not have to be selected *a priori*. Indeed, the learned weighting of features in a model can aid our mechanistic understanding of methylation. The authors of WHISTLE (whole-transcriptome m^6^A site prediction from multiple genomic features) showed that nucleotide sequence was the most important predictor of m^6^A but that 14 other genomic features also contributed. Among the top features was the site being in a long exon, which was previously found to be a defining characteristic of sites measured using m^6^A-CLIP (Dominissini et al., 2012; Ke et al., 2017). WHISTLE achieved an area under the curve of 0.948 when tested against previously unseen CLIP data.

Currently, all *in silico* m^6^A models use antibody-based methods as training data and so will also learn the biases present in them. To continue improving predictions, it will be important to generalize models by training on orthogonal datasets.

## Assembling a Dataset to Compare Detected and Predicted m^6^A Transcripts

**FIGURE 2:**
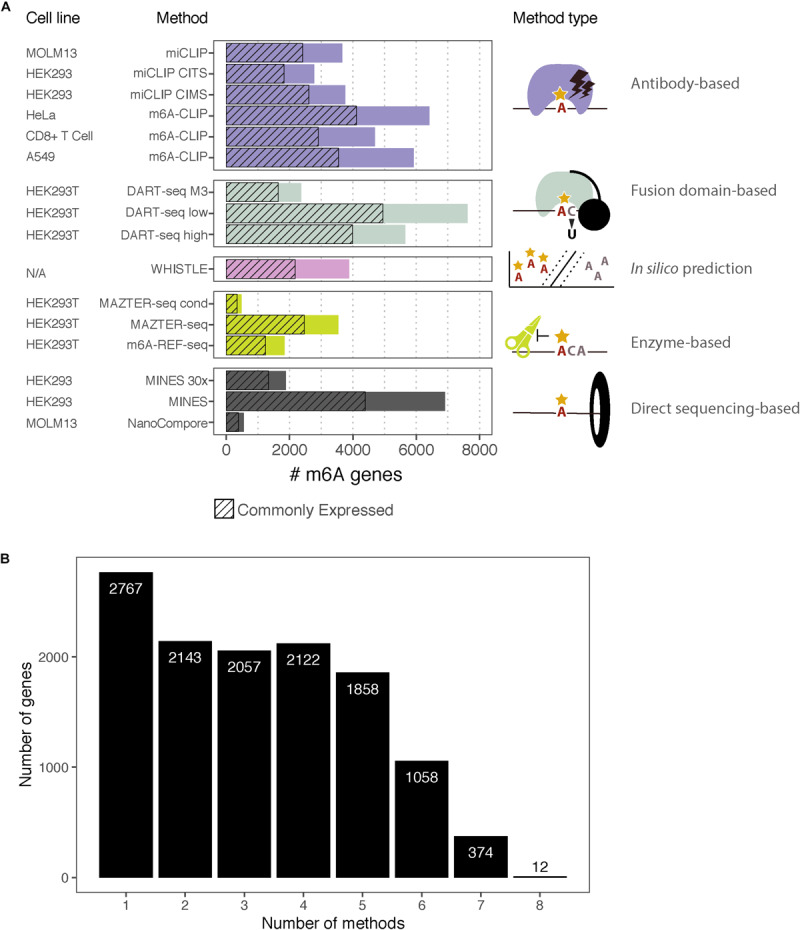
m^6^A-containing genes identified by eight methods. **(A)** Bar chart showing the number of m^6^A-containing transcripts identified by each method. Some methods have data from multiple cell lines or apply several possible thresholds, which are shown separately. The cell lines for each dataset are indicated along with the type of method. The hashed bars denote genes that are commonly expressed between all the cell lines considered here. For DART-Seq, MAZTER-Seq, and MINES, several thresholds were possible: “DART-Seq M3” refers to sites identified by comparison with METTL3 knockdown. “Low” and “high” refer to two stringency thresholds applied by the authors. “MAZTER-Seq” refers to all sites with a cleavage efficiency <50%, and “MAZTER-Seq cond” refers to FTO overexpression, WT ≥ 20%, and/or Alkbh5 overexpression, WT ≥ 20%. “MINES” refers to all sites identified by MINES, and “MINES 30×” refers to MINES sites with ≥ = 30× coverage. **(B)** Bar chart showing the numbers of overlapping target genes between the eight methods, considering all the reported genes.

The rapid expansion in orthogonal methods for transcriptomic m^6^A detection offers an opportunity to compare the published datasets. We assembled the processed data produced by eight high-resolution methods using human cells: two antibody-based CLIP approaches (miCLIP, m^6^A-CLIP); two endoribonuclease MazF-based (MAZTER-seq, m^6^A-REF-seq); one deamination approach (DART-seq); two using Nanopore direct RNA sequencing (MINES, NanoCompore); and finally, one based on computational predictions (WHISTLE). Here, we examine the overlap between these methods at the level of transcripts, focusing on a single representative transcript per gene. We include only sites with a matching DRACH motif, although some datasets have additional restrictions (such as MazF “ACA,” WHISTLE “RRACH,” and MINES “RGACH”). In total, we consider 134,470 unique sites in 12,391 mRNAs (Figures 2A,B; sites per gene are summarised in Supplementary Data Sheet S1).

### Filtering for Commonly Expressed Genes

Since there is not a single cell line that is used across all of the methods, we focused on commonly expressed mRNAs. For studies with no accompanying gene expression data, we accessed published RNA-seq measurements for equivalent cells lines from the EBI Expression Atlas (HEK293, HEK293T) and the Gene Expression Omnibus (MOLM13) (accession numbers listed in Table 2) (Edgar et al., 2002; Papatheodorou et al., 2018). For HEK293 and HEK293T, raw counts were assigned to the longest annotated transcript obtained from Ensembl BioMart v98 for GRCh38.p13, and transcripts per million (TPM) were calculated as expression measurements (Kinsella et al., 2011). For MOLM13 and HeLa, processed expression measurements were available as fragments per kilobase of transcript per million (FPKM) values. For A549 and CD8+ T cell, we used the matched poly-A sequencing data from the m^6^A-CLIP study. BedGraph files were downloaded, and coordinates were lifted over to hg19 using UCSC liftOver (Kuhn et al., 2013). Poly(A) sites were assigned to genes using bedtools closest -s -id -a stdin -b../hg19_mRNA_annotation.gtf -D a (Quinlan and Hall, 2010) with a threshold of 2,000 nt from the end of the annotated 3′ UTR. Expression was quantified as read counts per transcript. Expression values were visualized in histograms, with most cell lines displaying bimodal distributions allowing a straightforward separation of expressed and unexpressed genes. For A549 and CD8+ T cells, which displayed unimodal distributions, we applied an arbitrary threshold of five counts. Finally, for each cell type, we assigned expressed genes into deciles according to their expression values.

**TABLE 2:** Number of expressed genes per cell line and origin of the expression dataset.

**Cell line**	**Number of genes expressed**	**Accession**	**References**
HEK293	11,018	E-GEOD-44384 (EBI Expression Atlas)	Hussain et al., 2013
HEK293T	11,703	E-MTAB-7029 (EBI Expression Atlas)	Doumpas et al., 2019
MOLM13	12,968	GSE114111 (GEO)	Pei et al., 2018
HeLa	12,839	GSM2300445 (GEO)	Ke et al., 2017—m^6^A-CLIP paper
A549	9,963	GSM1828600 (GEO)	Ke et al., 2015—m^6^A-CLIP paper
CD8T+	8,235	GSM1828598 (GEO)	Ke et al., 2015—m^6^A-CLIP paper

**TABLE 3:** Number of m^6^A modified transcripts for each method following thresholding.

**Method**	**Sample**	**Thresholding**	**Number of transcripts**	**Number of total transcripts for method**	**Number transcripts** (**6,585 commonly expressed genes subset**)
miCLIP	CIMs HEK293	As from paper	3,755	6,282	4,000
	CITs HEK293	As from paper	2,779		
	MOLM13	As from paper	3,662		
m^6^A-CLIP	A549	As from paper	5,915	8,560	4,694
	CD8+ T cell	As from paper	4,697		
	HeLa	As from paper	6,415		
DART-seq	High stringency HEK293T	C > U events from paper filtered for DRACH motif	5,648	8,331	5,445
	Low stringency HEK293T	C > U events from paper filtered for DRACH motif	7,614		
	WT vs. METTL3 depleted HEK239T	C > U events from paper filtered for DRACH motif	2,370		
m^6^A-REF-seq	HEK293T	As from paper	1,843	1,843	1,243
MAZTER-seq	HEK293T	MazF cleavage efficiency < 50%	3,545	3,705	2,568
	HEK293T	FTO overexpression, WT ≥ 20%, and/or Alkbh5 overexpression, WT ≥ 20%	482		
WHISTLE	Trained on miCLIP and m^6^A-CLIP	Posterior probability of being m^6^A ≥ 0.95	3,877	3,877	2,177
MINES	Nanopore	As from paper	6,910	6,910	4,390
	Nanopore	Filtered for 30× coverage (threshold for NanoCompore)	1,883		
NanoCompore	WT vs. METTL3 KO Nanopore	DRACHs within clustered 5-mers with contextual *p* < 0.001	556	556	387

The procedure yielded between 8,235 and 12,968 expressed genes for each cell line (Table 2). Transcripts that were detected by the m^6^A measurement, but not RNA-seq, were assigned *post hoc* to the lowest expression decile of the cell line in question. In total, we considered 6,585 genes with commonly expressed transcripts across six cell lines.

### Comparison of the Top-Ranking Transcripts Between Methods

The eight m^6^A studies applied very different, and in some cases arbitrary, thresholds leading to large differences in the numbers of reported targets. In comparing the results, we found that studies reporting greater numbers of m^6^A targets tended to have better overlaps with other studies (data not shown), making them appear ostensibly more reliable; however, it is also possible that those methods suffer from higher false-positive rates.

To facilitate comparisons, we focused on the top ∼1,000 m^6^A modified transcripts for each method (Table 4). We wished to use “modification scores” for each study to identify thresholds that produce similar numbers of top-ranking targets; however, scores are not available for all methods, so instead, we ordered genes according to the number of detected m^6^A sites per transcript. NanoCompore reported only 387 transcripts that met our expression criteria, due to the lower sequencing throughput, the stringent requirement for 30× coverage over sites, and restriction to sites that change between wild type and METTL3 knockdown cells. In total, we considered 3,875 top-ranking transcripts among genes that are commonly expressed across all cell lines, with a total of 73,914 unique m^6^A sites.

**TABLE 4:** Number of top-ranking targets selected per method.

**Method**	**Number of transcripts**
DART-seq	1,019
m^6^A-CLIP	1,072
m^6^A-REF-seq	1,243
miCLIP	1,233
NanoCompore	387
WHISTLE	1,198
MINES	1,104
MAZTER-seq	944

**FIGURE 3:**
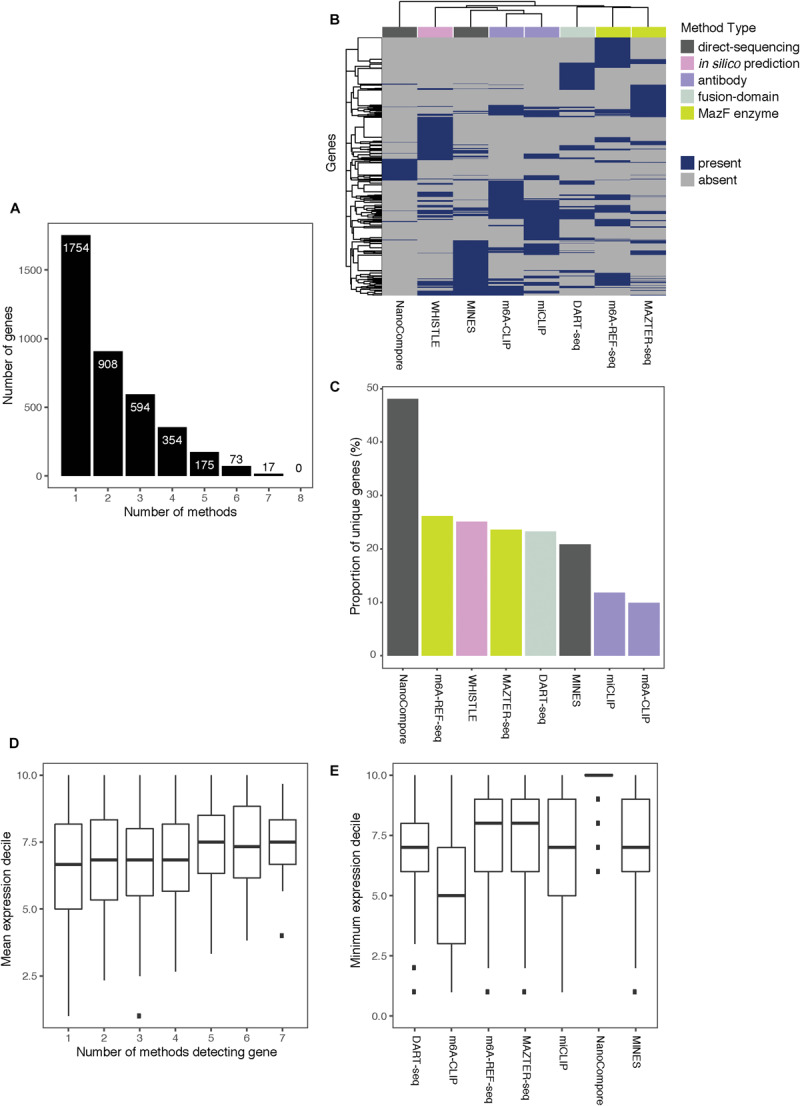
Comparing the top-ranking target genes identified by eight methods. **(A)** Bar chart showing the numbers of top-ranking genes that overlap between the eight methods. **(B)** Heatmap showing overlap between the top targets. Dendrograms are produced by complete-linkage hierarchical clustering using the Jaccard index as the distance metric. Dark blue indicates presence of the gene among the top targets for a method, and gray indicates absence. Colored bars denote the category of the method. **(C)** Proportions of top targets that are unique to each method. **(D)** Number of methods detecting a target gene plotted against its mean expression decile across all studied cell lines. **(E)** Minimum expression deciles for the top ranked genes were plotted for each method.

Of the 3,875 transcripts across all methods, 55% (2,121) are identified as m^6^A modified by at least two, 31% (1,213) by at least three, and 16% (619) by four or more methods (Figure 3A). Hierarchical clustering shows that methods of the same type cluster together, indicating that they are more likely to detect similar targets (Figure 3B); however, the shallowness of the dendrogram highlights that despite this, distinct methods tend to differ greatly in their outputs. WHISTLE and MINES cluster with the CLIP-based methods, reflecting the underlying training datasets. MAZTER-seq and m^6^A-REF-seq also cluster but share little overlap (40% of MAZTER-seq sites and 33% of m^6^A-REF-seq sites overlapped with each other). The method with the highest proportion of unique genes is NanoCompore (48%), followed by m^6^A-REF-seq (26%). The method with the lowest proportion of unique genes is m^6^A-CLIP (10%), which suggests its sites could be the most reliable (Figure 3C).

In general, the higher the expression, the more likely a transcript is to be identified by multiple methods (Figure 3D); this is expected as most of the experimental methods described here are biased toward highly expressed genes. In this regard, NanoCompore displays the largest expression dependence (Figure 3E). Interestingly, miCLIP shows a greater preference for highly expressed genes compared with m^6^A-CLIP, perhaps due to differences in starting RNA/antibody ratios in the immunoprecipitation step. In conclusion, the low overlap between methods may arise partly from the expression-linked bias in m^6^A detection and additional technical aspects of each method leading to different subsets of DRACH sites being detected.

## Discussion

Our analysis suggests that data coverage and mRNA expression are among the main biases for m^6^A detection. With sufficient coverage, potential sites of m^6^A modification can be detected in most mRNAs. However, in the absence of a gold standard, it is not possible at this point to estimate the false-positive rate of any single method for m^6^A detection nor of integrated datasets. This will be important moving forward because it is clear that different studies display varying degrees of overlap. Determining the reasons behind this is valuable for the community, especially as several databases now give users access to repositories of miCLIP data (CVm^6^A—Han et al., 2019; m^6^AVar—Zheng et al., 2017) and algorithms trained on such data are being used to make conclusions about the functionality and disease relevance of m^6^A sites (m^6^AVar—Zheng et al., 2017; Deep-m^6^A—Zhang S.-Y. et al., 2019; m^6^Acomet—Wu et al., 2019; DeepM^6^ASeq—Zhang and Hamada, 2018). Predictions will be limited by the validity of the training data, and it will be interesting to see how data from the newer non-antibody-based methods can be incorporated into such efforts.

In this review article, we performed analyses at the gene level as a tentative step to give the reader a broad perspective of the data types that are available for studies of m^6^A RNA modifications. An important aspect for further analyses will be to compare individual sites within a transcript across methods, experimental conditions, and variants of DRACH motif. In this way, it will be possible to address the positional or sequence biases of methods, compare the dynamics of m^6^A sites between conditions, cells or cellular compartments, and assess the modification rates of different DRACH sites. Such analysis could be approached in various ways, taking into account variable distances between sites assigned by different techniques and other method-specific issues. For such analyses, the use of unique molecular identifiers (UMIs) that control for PCR biases in library preparation—integrated into CLIP-based approaches—are particularly valuable. None of the antibody-free approaches currently use UMIs; therefore, quantifications of MazF and DART-seq datasets may be affected by variable PCR duplication rates. Direct RNA sequencing with Nanopores is not affected by PCR duplication, but the shallow sequencing depth may limit quantitative comparisons across large numbers of sites.

Finally, we have examined only m^6^A sites that occur within DRACH motifs, in line with the computational approaches used in past studies. In the future, it will be interesting to analyze noncanonical sites: currently, the technical noise is often too high to reliably include such sites and therefore appropriate controls will be needed, such as METTL3 depletion. This would also help establish the methylation status of lowly expressed genes, which generally have lower sequencing coverage.

Ultimately, untangling the benefits and biases of each method in determining m^6^A sites is crucial for the field as we move toward further understanding the mechanism, regulation, and function of m^6^A methylation on a transcriptomic scale.

## Author Contributions

JU, NL, and CC conceptualized the work. CC curated and analyzed the data and produced all tables and figures. CC, JU, and PT-K wrote the initial draft, with review and editing from NL. JU and NL supervised the work. The manuscript was finalized with input from all authors.

## Conflict of Interest

The authors declare that the research was conducted in the absence of any commercial or financial relationships that could be construed as a potential conflict of interest.
